# Novel Rotational Combination Regimen of Skin Topicals Improves Facial Photoaging: Efficacy Demonstrated in Double-Blinded Clinical Trials and Laboratory Validation

**DOI:** 10.3389/fmed.2021.724344

**Published:** 2021-09-17

**Authors:** Lisa DiNatale, Jolanta Idkowiak-Baldys, Young Zhuang, Anthony Gonzalez, Thomas J. Stephens, Lily I. Jiang, Weiping Li, Rubinder Basson, Ardeshir Bayat

**Affiliations:** ^1^Global Innovation Center, Avon Products Inc., Avon Skin Care Institute, Suffern, NY, United States; ^2^Thomas J. Stephens & Associates, Inc., Richardson, TX, United States; ^3^Centre for Dermatology Research, National Institute for Health Research Manchester Biomedical Research Centre, Stopford Building, University of Manchester, Manchester, United Kingdom; ^4^Medical Research Council of South Africa Wound Healing Unit, Hair and Skin Research Laboratory, Division of Dermatology, University of Cape Town, Cape Town, South Africa

**Keywords:** rotational combination regimen, skin cosmetics, skin topical application, facial photoaging, cosmetic dermatology

## Abstract

Topical antiaging products are often a first-line intervention to counter visible signs of facial photoaging, aiming for sustained cosmetic improvement. However, prolonged application of a single active topical compound was observed clinically to lead to a plateau effect in improving facial photoaging. In view of this, we set out to reduce this effect systematically using a multi-tiered approach with laboratory evidence and clinical trials. The objective of the study was to evaluate the effects of active topical ingredients applied either alone, in combination, or in a rotational manner on modulation of facial photoaging. The study methodology included *in vitro*, organotypic, and *ex vivo* skin explants; *in vivo* biopsy study; as well as clinical trials. We demonstrate for the first time that a pair of known antiaging ingredients applied rotationally, on human dermal fibroblasts, maximized pro-collagen I production. Indeed, rotational treatment with retinol and phytol/glycolic acid (PGA) resulted in better efficacy than application of each active ingredient alone as shown by explants and *in vivo* biopsy study, with penetration of active ingredients confirmed by Raman spectroscopy. Furthermore, two split-face, randomized, double-blinded clinical trials were conducted, one for 12 months to compare treated vs. untreated and the other for 6 months followed by a 2-month regression to compare treated vs. commercially marketed products. In both studies, rotational regimen showed superior results to its matching comparison as assessed by clinical grading and image analysis of crow's feet wrinkles. In conclusion, rotational regimen using retinol and PGA is effective in treating facial photoaging signs with long-lasting benefits.

## Introduction

**Figure 1:**
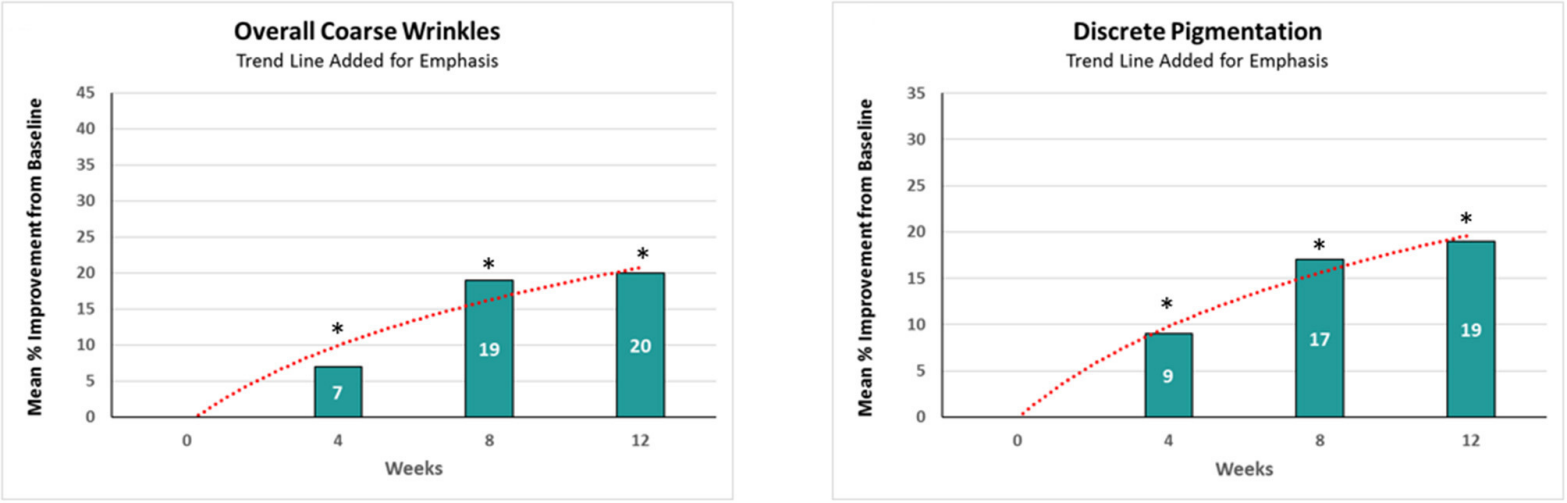
The effect of retinol on clinical efficacy rate of improvements. A significant (*p* < 0.05) improvement in both coarse wrinkles and discrete pigmentation was observed as early as 4 weeks, but the rate of improvement achieved over a given time interval decreased as the study progressed. *Means *p* < 0.05 or statistically significant result.

Chronic sun exposure, pollution, and intrinsic aging can add wrinkles and age spots to the facial skin (1–6). With consumers desiring healthier and younger-looking skin, there is a high demand for effective topical anti-aging cosmetic treatments with long-lasting results. Active ingredients such as alpha-hydroxy acids, antioxidants, and retinol can boost collagen production and diminish the appearance of wrinkles (7–11). Among these, retinol is recognized as one of the most effective (12, 13). However, there are limitations and certain side effects associated with continued use of retinol products alone (14). In addition, our clinical observations (*n* = 32) demonstrated that prolonged application of a single active ingredient led to a plateau effect in improving facial photoaging (Figure 1). Furthermore, feedback from consumers (own unpublished data) showed that, after using products for a few months, there appears to be a “plateau effect” in which the product stops improving their skin and signs of photoaging. Another efficacious ingredient with known mechanism of action complementary to retinol is glycolic acid (15). Combining them together could potentially lead to better rate of improvements. However, combining these two topicals can be challenging due to formulation requirements (unpublished own data). An alternative approach of maximizing retinol efficacy could be by using a phytanic acid precursor, phytol, which has a mechanism of action similar to retinol and complements it by barrier protecting properties.

**Figure 2:**
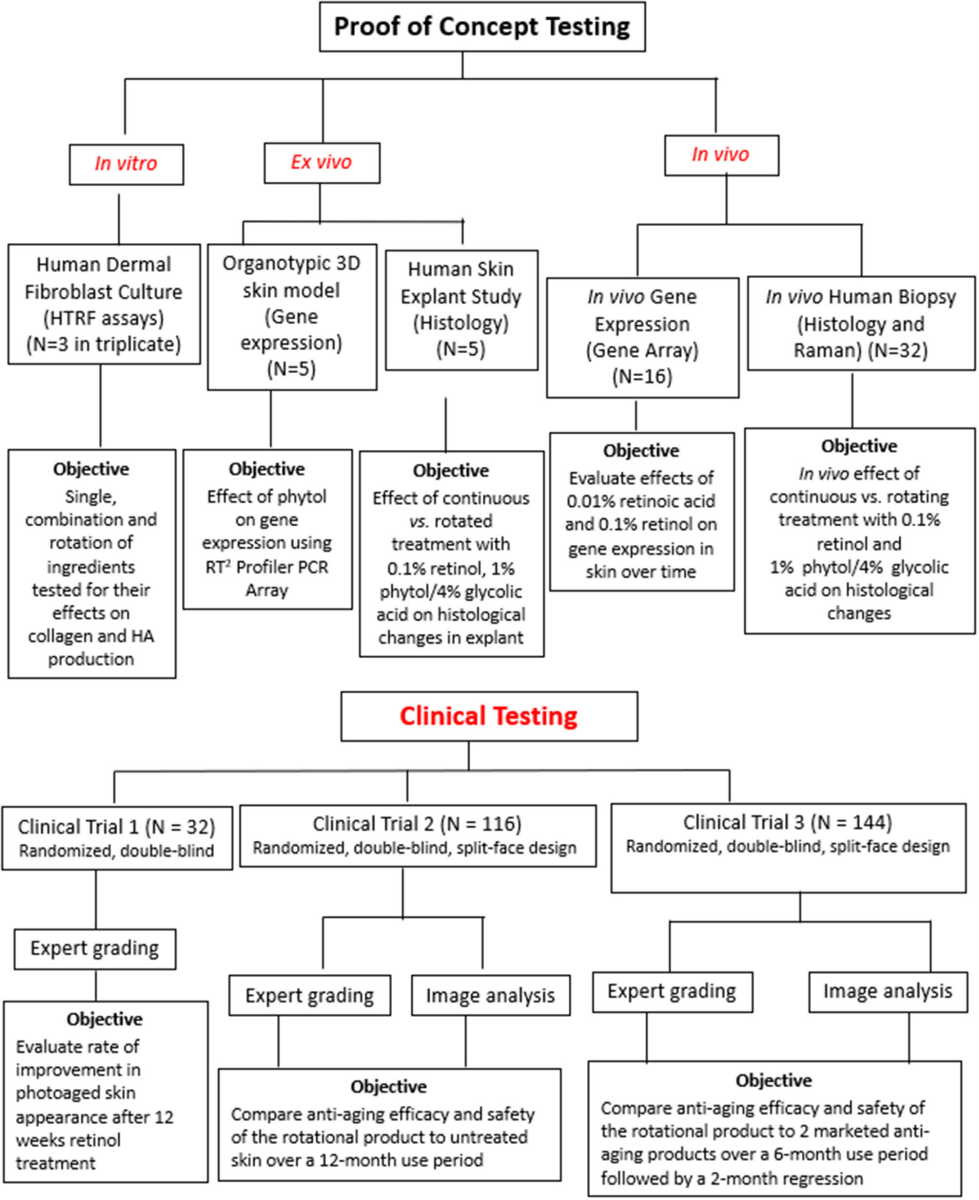
Study methodological flowchart. This flowchart represents the summary of laboratory and clinical testing conducted with the novel rotational regimen.

In view of the above, we set out to address this issue systematically (Figure 2). We examined the effect of previously proven single active ingredients using two different model systems, discussed below. These experiments confirmed that there was a plateau effect in response to a single active ingredient in isolation. Thus, we conducted a series of experiments to assess the effects of varying application regimens of active ingredients on the production of dermal matrix components using human dermal fibroblasts i*n vitro* and 3-dimensional (3D) organotypic skin models. Ingredients were tested alone, in combination, and in a rotational manner. These experiments identified that ingredients applied in rotation for a period of time resulted in greater pro-collagen I production compared with the continuous application of either ingredient alone for the same period of time. The results were further solidified with evidence from an *ex vivo* skin model using whole human skin organ culture and *in vivo* human volunteer biopsy studies. In addition, using Raman spectroscopy (RS), we confirmed penetration of the active ingredients and demonstrated structural changes in the skin following application of the active ingredients in each layer of the skin.

Finally, two split-face, randomized, institutional review board (IRB)-approved, double-blinded clinical trials were conducted to compare the efficacy of this novel rotational regimen vs. an untreated condition. Treatments were also compared against commercially comparable marketed products.

## Materials and Methods

### *In vitro* Study Using Cultured Human Dermal Fibroblast Cells

Human dermal fibroblast cells were grown in a six-well plate in Dulbecco's Modified Eagle Medium (DMEM) (Corning, USA) supplemented with 10% fetal bovine serum (FBS) and L-glutamine (1.5 × 10^5^ cells/plate). After reaching about 75% confluence, cells were transferred into DMEM without FBS and incubated for 4–6 h. Next, cells were treated with given active ingredients in DMEM without FBS. Media were collected and treatment was repeated every 48 h. Cells were either treated with a single ingredient over the entire course of treatment, treated with a combination of ingredients, or treated with one ingredient for 6 days followed by treatment with alternative ingredients for the remaining time. After treatment, conditioned media were collected, and the amount of collagen and hyaluronic acid secreted was tested in the media using Homogeneous Time Resolved Fluorescence (HTRF) human pro-collagen I or hyaluronic acid binding protein assay kit (Cisbio Inc., Bedford, MA, USA).

### Phytol Inhibition of Tumor Necrosis Factor α-Induced NFKB Phosphorylation Assay

Human epidermal keratinocytes were grown in six-well plates (*n* = 3 for each treatment). The next day, cells were pretreated with phytol for 24 h. After the pretreatment, 100 ng/ml tumor necrosis factor (TNFα) was added for 5 min to induce the nuclear factor kappa-light-chain-enhancer of activated B cells (NFκB) pathway. After treatment, cells were washed with phosphate-buffered saline (PBS), and lysis buffer was added directly to cells followed by freezing at −80°C. Cell lysates were normalized to the same protein concentration, and NFκB phosphoS536 was measured using HTRF technology (Cisbio Inc., Bedford, MA, USA).

### Gene Expression Analysis

Sixteen Caucasian post-menopausal female subjects between 55 and 65 years of age with skin types II–III were recruited for the study. The dorsal forearms of the subjects were patch treated with either 0.01% retinoic acid (RA) or 0.1% retinol (daily for the first 5 days, alternate days subsequently). On days 4, 8, 15, and 30, 3-mm punch biopsies were taken from the treated and adjacent untreated areas (with approval of an IRB and informed consent of the subjects). RNA from the biopsy samples were isolated using the RNeasy method (Qiagen, UK). RNA samples were converted into labeled target antisense RNA (cRNA) using the Single-round RNA amplification and biotin labeling system (Enzo). The resulting cRNA is fragmented and applied to Human U133 Plus 2.0 arrays. Following hybridization, arrays were washed and stained using standard Affymetrix procedures before scanning on the Affymetrix GeneChip Scanner and data extraction using Expression Console. Pathway analysis was conducted using Ingenuity Systems (Qiagen, Redwood City, USA).

### 3D Organotypic Tissues and *ex vivo* Studies

3D skin model EPI200 (MatTek, Ashland, USA) was maintained in media supplied by manufacturer. Onto the surface of tissues, 1% phytol was applied in five replicates. After 24 h of treatment, tissues were rinsed and fresh treatment was added for additional 24 h, for total of 48-h incubation. After treatment, tissues were rinsed and frozen in liquid nitrogen for RNA isolation. RNA was extracted from tissues using TRIzol reagent (Thermo Fisher Scientific Inc., Waltham, USA). Total RNA was used for cDNA synthesis using RT^2^ First Strand Kit (Qiagen; Valencia, USA). The expression of genes highly relevant to skin biology was analyzed using custom-designed RT^2^ Profiler PCR Array (Qiagen, UK).

*Ex vivo* analysis was performed on surgically removed skin with written informed consent and with ethical and institutional approval from the University of Manchester (ethics reference: 11/NW/0683). All tissues were tracked and stored in a human tissue biobank following guidelines set by the Human Tissue Act. To a 24-well plate containing trans-well inserts, 6-mm punch biopsies of skin were added, allowing the biopsy to be suspended in liquid culture media while keeping the epidermis separate and exposed to air for the addition of treatments. To each well, 500 μl of supplemented serum-free William's E culture media (1% penicillin/streptomycin, 1% L-glutamine, 1% non-essential amino acid solution, 1% ITS+3, and 10 ng/ml hydrocortisone) was added and changed daily. Antibiotics were used in order to prevent contamination by the microflora naturally present on human skin. The culture model was maintained in an incubator under standard conditions (37°C, 5% CO_2_). Skin explants were treated with two formulations, one containing retinol and one phytol/glycolic-based, either alone or in sequence rotation. Treatments were repeated daily for 12 days. For continuous treatment, samples were treated with a formulation containing either 0.1% retinol or 4% glycolic acid and 1% phytol alone. For rotational treatment, samples were treated with a formulation containing 4% glycolic acid and 1% phytol for 3 days, followed by a formulation containing 0.1% retinol for 3 days, with a total of two rotation cycles. After treatment, samples were fixed in formalin and processed for histological analysis following standard protocol.

### *In vivo* Biopsy Collection

The study was conducted at Product Investigations, Inc. (Conshohocken, USA) following a standard protocol. Briefly, Caucasian female (*n* = 32) subjects between 21 and 70 years of age participated in an application regimen over a 4-week period. The lower volar arms of subjects were treated with test articles for 4 weeks under patch. Treatments were repeated daily and remained under patch over the weekend. For continuous treatment, subjects were treated with a formulation containing either 0.1% retinol or 4% glycolic acid and 1% phytol (PGA). For “rotational” treatment, subjects were treated with a formulation containing PGA on weeks 1 and 3, while a formulation containing 0.1% retinol was used on weeks 2 and 4. After the treatment, a sample of the skin was obtained *via* biopsy (2 mm) from each of the test/control sites. Skin samples were analyzed by histology and Raman spectroscopy.

### Histological Analysis

The skin samples were fixed in 10% formalin. Next, samples were sequentially dehydrated in 70, 95, and 100% ethanol; cleared in xylene; and embedded in paraffin. Following this, 6-μm sections were obtained using a microtome and processed for histological analyses. Hematoxylin and eosin (H&E) staining was performed as per standard H&E protocol, and slides were scanned by 3DHistech Pannoramic-250 microscope slide scanner. Three measurements of the cellular portion of the epidermal compartment were performed using CaseViewer2.2. Subsequently, immunohistochemistry was conducted according to the following: sections were dewaxed in xylene followed by rehydration. Then, heat-induced antigen retrieval was performed with sections incubated in pre-boiled citrate buffer (pH 6.0 × 10) in a microwavable vessel at 80-W power for 20 min then cooled for 30 min at room temperature. Following incubation in BLOXALL Endogenous Peroxidase and Alkaline Phosphatase Blocking Solution (Vector Laboratories, UK) for 10 min. Sections were incubated with biotinylated hyaluronic acid-binding protein (HABP) (MERCK cat 385911) antibody at 0.5 μg/ml in DAKO antibody diluent (S0809) overnight at 4°C. After incubation with an AP detection system (VECTASTAIN ABC-AP Staining Kit VECTOR AK 5000) for 30 min, sections were rinsed well with PBS, then incubated with Vector Blue Alkaline Phosphatase Substrate (VECTOR SK5300) for 20 min. The reaction was completed, and slides were mounted with an aqueous mount medium. For Herovici staining, the slides were incubated in Van Gieson stain solution (50% Van Gieson solution, Sigma HT254-250ML; 0.025% methyl blue, Sigma cat M44907; 10% glycerol, Fisher Chemical cat G/0650/08; and 0.5% lithium carbonate, Fluka cat 62472) for 10 min, then differentiated in 1% acetic acid for 30 s, dehydrated through alcohol and xylene, and mounted. The sections were scanned and analyzed using Definiens Tissue Studio software, version 64.4.0 (Definiens, Munich, Germany) and MATLAB software.

### Raman Spectroscopy

Skin biopsies of 2-mm diameter were snap frozen, cut into 10-μm cross-sections, and placed onto Raman-grade calcium fluoride slides (Crystran Ltd, UK). The cross-sections were analyzed on a Renishaw inVia Raman microscope (Renishaw, UK), using an optimal wavelength of 785 nm. Samples were visualized and each dermal layer identified (epidermis, papillary, and reticular dermis). Three static spectral point measurements were taken in each layer using WiRE (Windows-based Raman Environment; Renishaw, UK). Optimized conditions required 100% laser power, for 10-s exposure time and 20 accumulations (a total measurement time of 200 s per point); prior to each measurement, a depth series acquisition was undertaken to ensure the sample was flat and to optimize penetration depth. After acquiring each measurement, cosmic ray removal was performed. Data analysis was undertaken in MATLAB (MathWorks, USA), using Renishaw's MATLAB plugin scripts to allow for statistical analysis.

### Clinical Study

#### Subject Selection

Eligible women aged 35–72 years in good health (neither pregnant nor lactating as assessed *via* questionnaire) were recruited and selected. Subjects were skin types I–IV who presented mild to moderate signs of photodamage (wrinkles, hyperpigmentation, and lack of radiance) on both sides of their faces and scored 3–6 to qualify on a 0–9 scale (modified Griffiths scale 0–9: 0 = none, 1–3 = mild, 4–6 = moderate, and 7–9 = severe).

#### Trial Design

**Figure 3:**
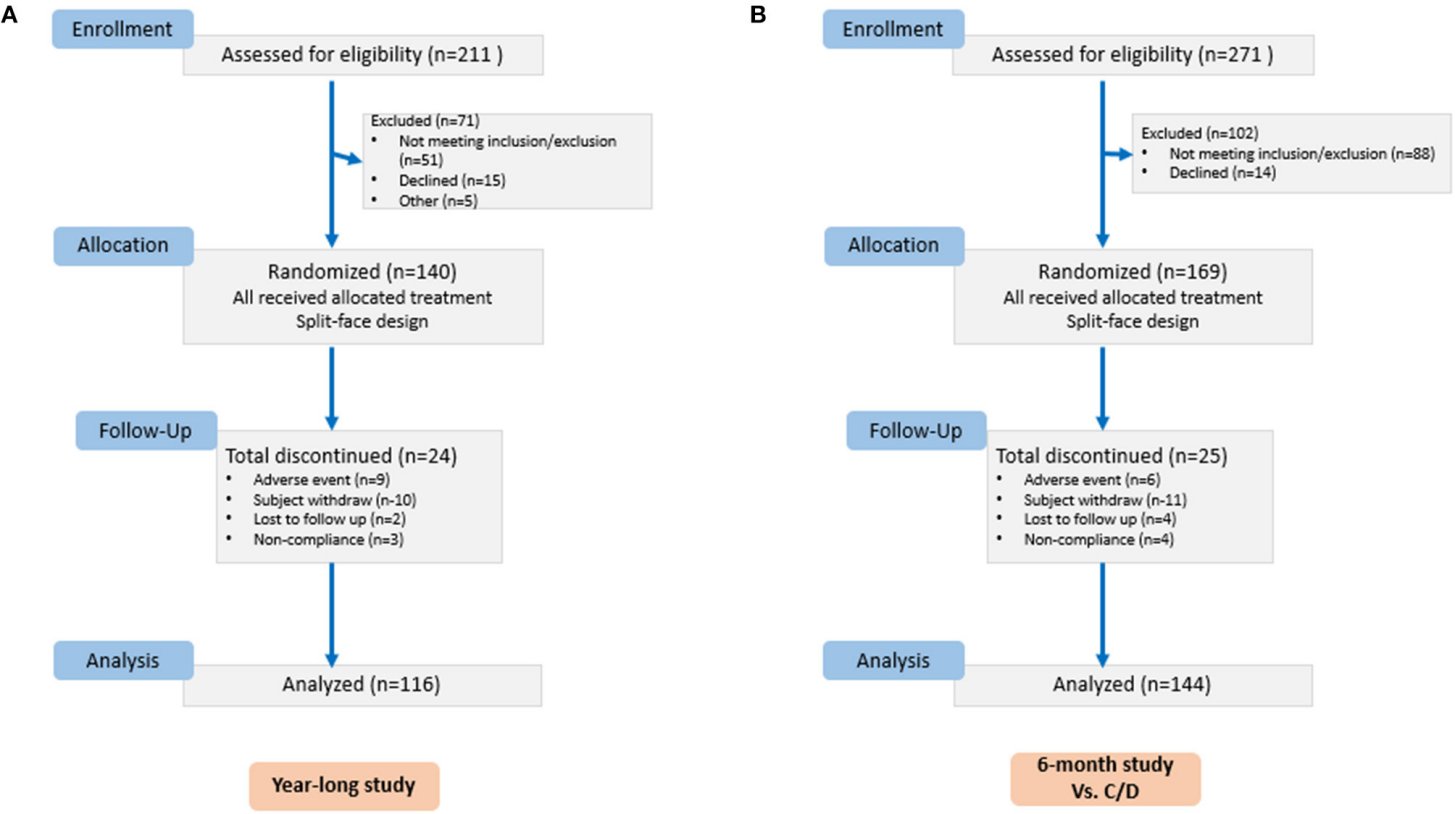
Study details of clinical trial #2 **(A)** and #3 **(B)**.

Clinical studies were divided into three independent trials. The first trial was conducted to assess the rate of improvement after retinol treatment. The second trial assessed the efficacy of rotational treatment over 1-year period. The third trial compared the rotational treatment formulation to two commercially available antiaging products. Trial details can be found in the CONSORT flowchart (Figure 3).

The first trial was conducted on 32 healthy females aged 35–59 who were enrolled with moderate (grades 3–6) wrinkles. Subjects had mild to moderate grades for other signs of photodamage. The second trial was conducted in 116 healthy female volunteers, aged 35–72, for 12 months. The subjects were randomized and assigned to use the regimen on the left or right side of their faces with the other side untreated. The third study was conducted in 144 healthy female volunteers with 6 months of product usage followed by a 2-month regression period. All clinical trials were conducted at Stephens & Associates in Texas and received IRB approval by IntegReview IRB. Subjects were randomized and assigned to the regimen on the left or right side of their faces. In the third study, subjects were randomized into one of two groups at the baseline visit. Group 1 subjects used the rotational products on one side of their faces and used a commercial product on the other side. In group 2, subjects used the rotational products on one side of their faces and used a different commercial product on the other side. Study participants gave a written informed consent. Trials followed the Declaration of Helsinki and were registered with ISRCTN (ISRCTN17303898, May 2020).

In the first study, subjects applied 0.1% retinol to their faces once daily for 12 weeks; a standard cleanser and sunscreen were provided. A dermatologist evaluated subjects' skin condition at baseline and after 4, 8, and 12 weeks. In the second study, at the baseline visit, eligible subjects were randomized to use the rotational product [alternating weekly between treatment A (PGA) and treatment B (containing retinol)] on the left or right side of the face, with the other side of the face untreated. Subjects returned to the clinic at weeks 4, 8, 12, 18, 24, 36, and 52 for clinical evaluation and digital imaging procedures. In the third study, subjects were randomized into one of two groups at the baseline visit. Group 1 subjects used the rotational products on one side of their faces and used a commercial product (C) on the other side. In group 2, subjects used the rotational products on one side of their faces and used a commercial product (D) on the other side. Clinical evaluations and digital imaging procedures were conducted at baseline and weeks 2, 4, 8, 12, 18, 24, and 32.

#### Trial Assessment

Clinical evaluations on facial photoaging signs were done by trained clinical evaluators using a modified Griffiths scale (0–9: 0 = none, 1–3 = mild, 4–6 = moderate, and 7–9 = severe) (16). The following parameters were evaluated for each side of the face: fine lines and coarse wrinkles (global face and crow's feet area), hyperpigmentation, skin tone (color) evenness, tactile texture, visual texture, clarity, radiance, firmness (tactile), and sagging. Safety assessment on tolerability was assessed on a 0–3 scale (0 = none, 1 = mild, 2 = moderate, and 3 = severe) for erythema, dryness, edema, burning, itching, and stinging. Digital photographs were taken using a photo station with a Nikon D7000 digital SLR camera under raking light, cross-polarized light, and visible light. Crow's feet wrinkles and lines were quantified using the SWIRL method (17).

### Statistical Analysis

The software used was SAS version 9.4 (SAS Institute, Care, NC, USA). Clinical grade and image data for treated sides of the face, adjusted for the untreated side, were analyzed by ANOVA (treated–untreated) or ANCOVA (untreated as covariate) for clinical study 1 and, where appropriate, Tukey's test was used to compare group means. A two-tailed significance level of α = 0.05 was used for all statistical comparisons. Raman spectroscopy data was tested by a one-way ANOVA. The data were parametric, and therefore *t*-test was used for assessment of statistical significance.

## Results

### Efficacy of a Single Active Ingredient Plateaus Over Time

**Figure 4:**
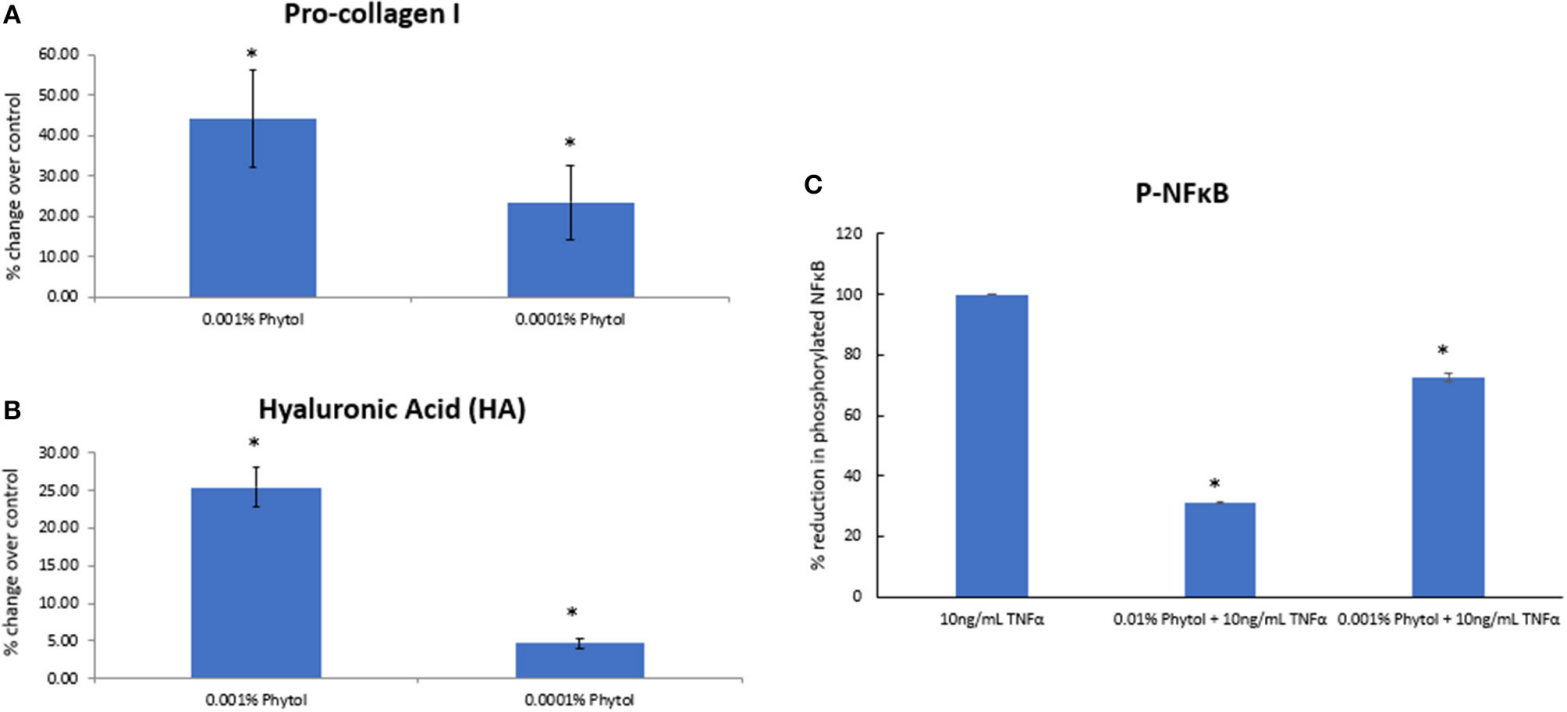
The effect of phytol on pro-collagen I, hyaluronic acid (HA) production in human dermal fibroblasts, and nuclear factor kappa-light-chain-enhancer of activated B cells (NFκB) phosphorylation in human epidermal keratinocytes. Phytol increased the levels of pro-collagen-I **(A)** and hyaluronic acid **(B)** in human primary dermal fibroblasts following 48-h treatment (*p* < 0.05). **(C)** Phytol reduced the levels of phosphorylated NFκB in tumor necrosis factor α (TNFα)-treated human primary keratinocytes in a dose-dependent manner (*p* < 0.05). The assays were performed in triplicate for each sample, and the values represent mean ± SD (standard deviation). *Means *p* < 0.05 or statistically significant result.

**Figure 5:**
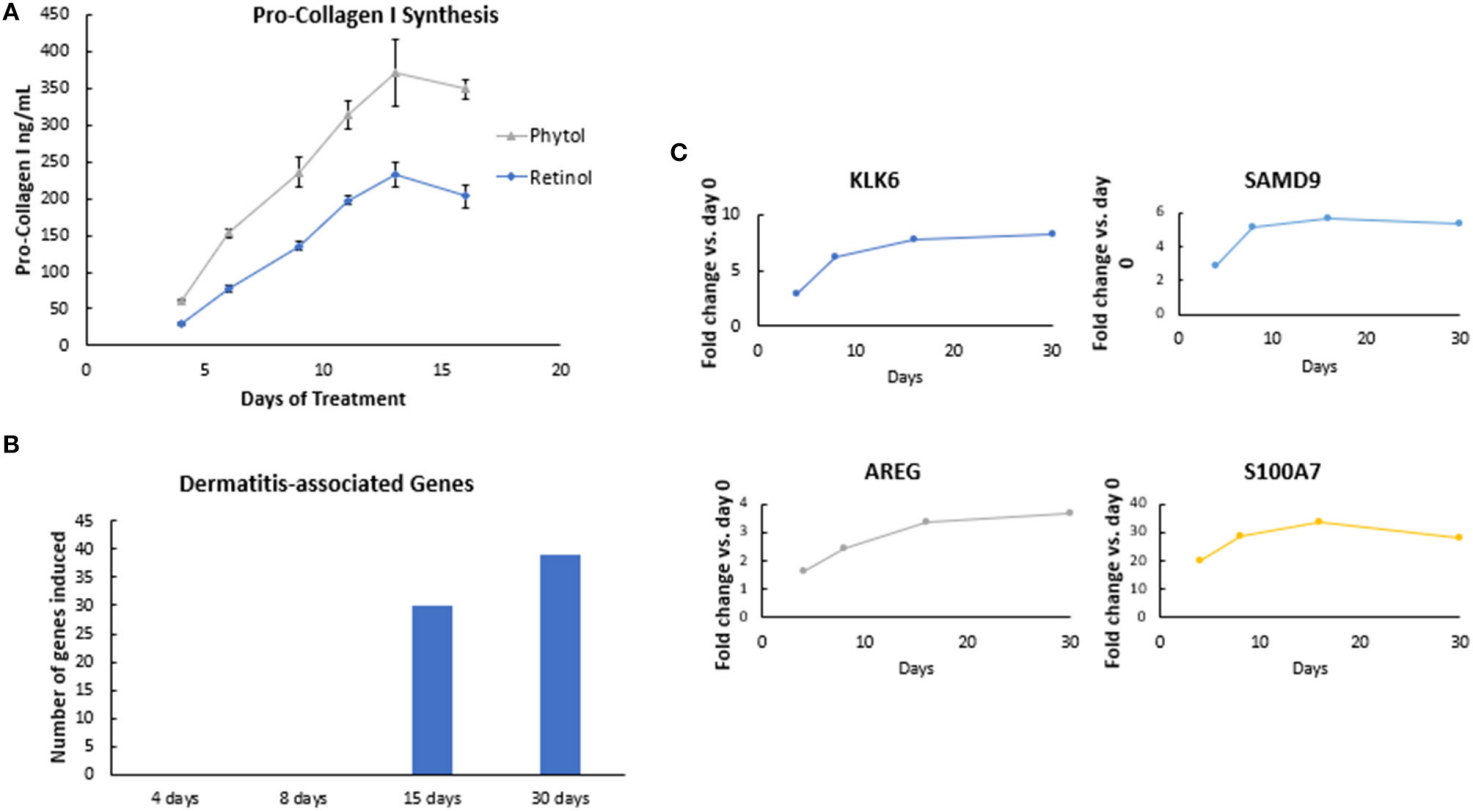
Efficacy rate evaluation. **(A)** The effect of retinol and phytol on pro-collagen I synthesis in human dermal fibroblast cells. Human dermal fibroblasts were treated with 0.0001% phytol or 0.00003% retinol for a period of 16 days, and production of pro-collagen I was measured. The assay was performed in triplicate for each sample, and the values represent mean ± SD. Stimulation of pro-collagen I synthesis was observed with both active ingredients (*p* < 0.05), but the rate of improvements decreased over time. **(B)** The effect of retinoic acid on gene expression in human skin. A formulation containing 0.02% retinoic acid was applied under occlusion on the lower dorsal arm. Subjects were treated daily for the first 5 days followed by treatment on alternate days for a total of 30 days. Skin samples were collected by punch biopsy on days 4, 8, 15, and 30. RNA was extracted, and whole genome expression was analyzed by Human U133 Plus 2.0 arrays. Retinoic acid treatment resulted in upregulation of Kallikrein-6 (KLK6), Sterile alpha motif domain-containing protein 9 (SAMD9), Amphiregulin (AREG), and Psoriasin (S100A) genes, which peaked on days 4–8, *p* < 0.05. **(C)** The effect of retinol on dermatitis-associated genes in human skin. A formulation containing 0.1% retinol was applied under occlusion on the lower dorsal arm. Subjects were treated daily for the first 5 days followed by treatment on alternate days for a total of 30 days. Skin samples were collected by punch biopsy on days 4, 8, 15, and 30. RNA was extracted, and whole genome expression was analyzed by Human U133 Plus 2.0 arrays. Pathway analysis performed using Ingenuity Pathway Analyses (IPA) showed that there was an increase in the number of dermatitis-associated genes after continued retinol treatment detectable at day 15.

Retinol and glycolic acid are widely accepted as efficacious antiaging ingredients (15, 18), while phytol shows strong potency *in vitro* on induction of dermal matrix components, such as pro-collagen I and hyaluronic acid (HA) inhibition of pro-inflammatory signaling (NFκB) (Figure 4), and modulation of genes related to epidermal turnover, skin barrier, inflammation, and matrix remodeling (Table 1). When skin cells are exposed to retinol or phytol, both ingredients are converted to their bioactive forms, namely, retinoic acid and phytanic acid, which are known to bind to their respective receptors and induce downstream signaling (19, 20). Human dermal fibroblasts were treated with 0.0001% phytol or 0.00003% retinol for an extended period (16 days), and production of pro-collagen I, a marker of antiaging that correlates with improvements in wrinkles (21, 22), was measured (21, 22). Stimulation of pro-collagen I synthesis with both active ingredients decreased over time and the effect leveled off (Figure 5A).

**Table 1:** Phytol effect on skin-related genes in 3D epidermal skin equivalent (EPI200).

**Gene**	**Fold regulation**
FN1	3.61
KLK6	2.27
HBEGF	2.23
TNC	2.01
LOR	1.83
ALOX12B	1.48
HAS3	1.44
ALOX12	1.44
PTGS2	−1.22
MMP3	−3.11

*Results are shown as up-down regulation compared to control group. Phytol significantly (p < 0.05) increased gene expression of Fibronectin 1 (FN1), Kallikrein-6 (KLK6), Heparin-Binding Epidermal Growth Factor-Like Growth Factor (HBEFG), Tenascin C (TNC), Loricrin (LOR), Arachidonate 12-lipoxygenase B (ALOX12B), Hyaluronan synthase 3 (HAS3), and Arachidonate 12-lipoxygenase (ALOX12). Phytol significantly (p < 0.05) decreased the gene expression of prostaglandin-endoperoxide synthase 2 (PTGS2) and matrix metalloproteinase-3 (MMP3)*.

Next, we investigated the rate of retinol efficacy by gene expression analysis using an *in vivo* human volunteer biopsy study (*n* = 16). After treatment with formulations containing 0.02% retinoic acid or 0.1% retinol, 2-mm punch biopsies were taken on days 4, 8, 15, and 30 from the volunteers' forearms. Volunteers were treated daily for the first 5 days followed by treatment on alternate days for a total of 30 days. Whole genome expression microarray analysis (Human U133 Plus 2.0 arrays) demonstrated upregulation of several retinoic acid-induced genes (such as KLK6, SAMD9, AREG, and S100A), which peaked on days 4–8 (*p* < 0.05), then leveled off over time (Figure 5B), confirming our initial observation of the plateau effect from the *in vitro* experiment. Furthermore, Ingenuity Pathway Analyses (IPA) of the retinol gene array data showed that there was an increase in the number of dermatitis-associated genes over time after continued retinol treatment. IPA analysis demonstrated collective gene expression changes with an increase in dermatitis on days 15 and 30 (Figure 5C). Taken together, these results further emphasize the suboptimal response of using one active ingredient in isolation over a continued period of time.

### Rotational Application of Active Ingredients *in vitro* Enhances Efficacy

Thus, it was hypothesized that the observed plateau effect could be prevented by “rotational” application of active ingredients that would lead to stimulation of different biological pathways in skin. Human dermal fibroblasts were treated with either phytol or retinol continuously for 12 days, a combination of both, or in rotation (6 days of treatment with phytol followed by 6 days of treatment with retinol). Rotational treatment led to an increase in pro-collagen I production (*p* < 0.05) compared to either of the active ingredients alone (Table 2). In view of this, we focused our attention on a rotational regimen of these two active ingredients. In addition, in order to gain maximum clinical efficacy, we combined phytol with glycolic acid.

**Table 2:** The effect of active ingredient application on pro-collagen I in human dermal fibroblasts *in vitro*.

**Ingredient A**	**Ingredient B**	**A**	**B**	**A+B combined**	**A/B rotation**	**B/A rotation**
		**% change in pro-collagen I production**				
Phytol 0.0001%	Retinol 0.00003%	19.1	3.9	7.09	75.7^*^	92.6^*^

*Rotational treatment led to a significant (p < 0.05) increase in pro-collagen I production compared to other treatments*.

* *Means p < 0.05 or statistically significant result*.

### *Ex vivo* Analysis of Rotational Regimen Demonstrates Enhanced Efficacy

**Figure 6:**
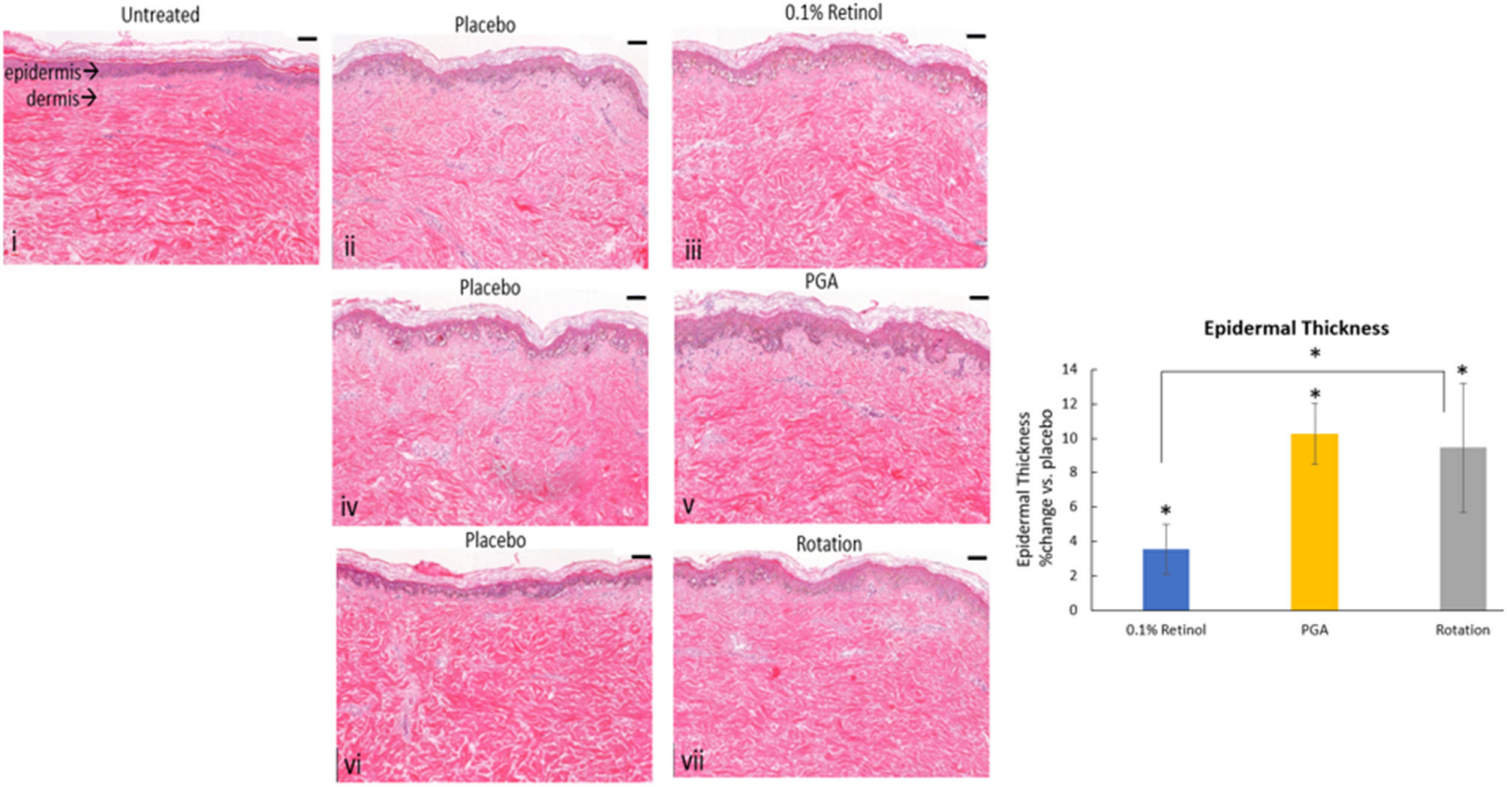
The effect of rotational treatment on epidermal thickness in skin explants *ex vivo*. Skin explants (*n* = 5) were treated (in triplicate) with continuous application of a single formula for 12 days or with two rounds of rotational application of each formula (per 3 days). Epidermal thickness was assessed by hematoxylin and eosin (H&E). All treatments increased epidermal thickness when compared to a placebo (*p* < 0.05), while rotational treatment was significantly better than retinol formula alone. i: untreated, ii: placebo for retinol, iii: retinol, iv: placebo for 1% phytol/4% glycolic acid (PGA), v: PGA, vi: placebo for vii, and vii: rotation of iii and v. Scale bar = 100 μm. *Means *p* < 0.05 or statistically significant result.

Topical application of formulas containing 0.1% retinol or 1% phytol formulated with 4% glycolic acid (PGA) was assessed on *ex vivo* skin samples (*n* = 5). H&E staining (Figure 6) evaluation showed a significant effect of all formulations (*p* < 0.01) on increasing epidermal thickness when compared to a placebo. Interestingly, rotational treatment was comparable to PGA and significantly better than retinol formula alone.

### *In vivo* Histological Analysis of Phytol/Glycolic Acid and Retinol Confirms the Role of Rotational Application

**Figure 7:**
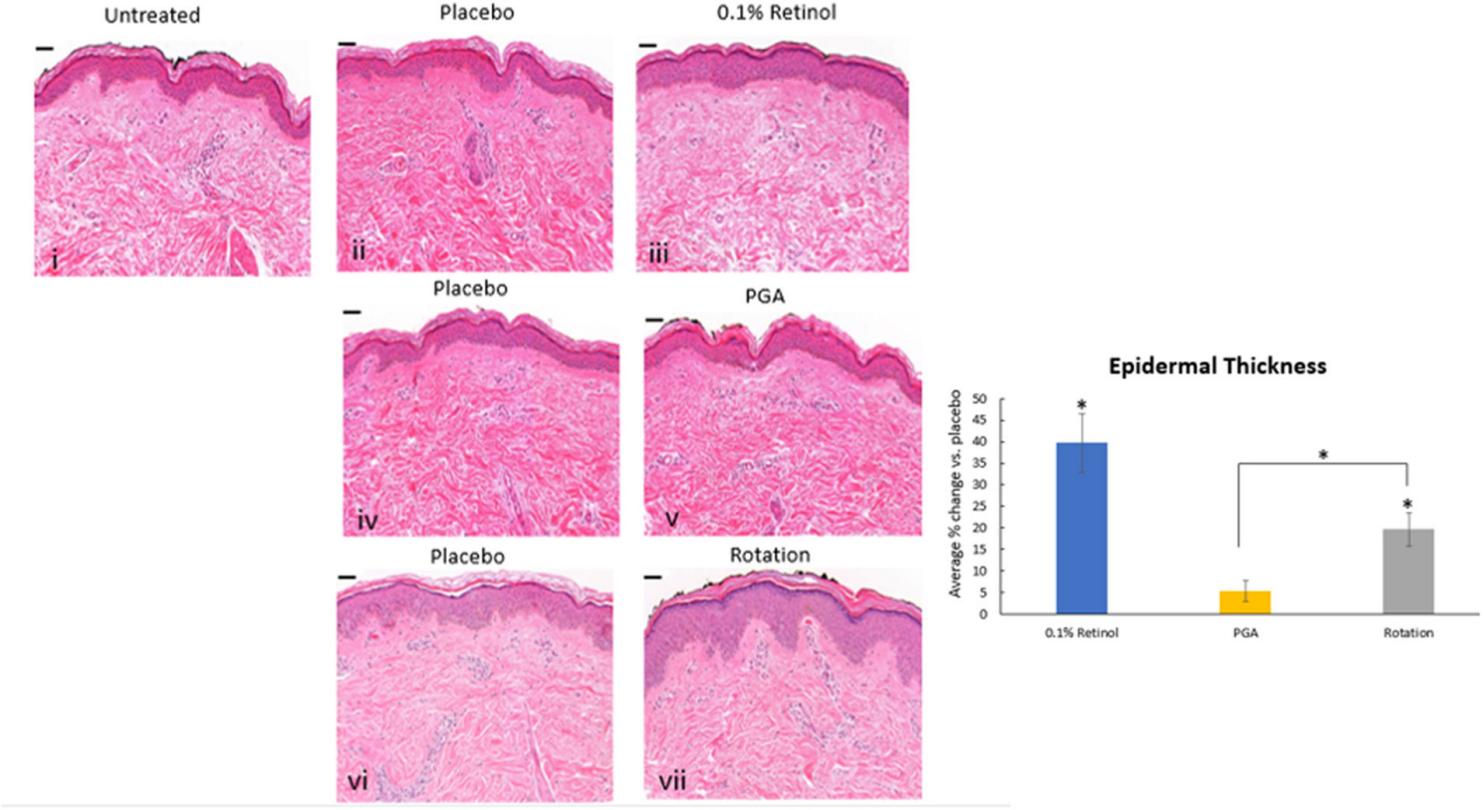
Histology of skin biopsies. Volunteers (*n* = 32) received application of either 0.1% retinol formulation for 4 weeks or weekly rotation of 1% phytol/4% glycolic acid (PGA) formula with 0.1% retinol formula. Biopsy samples were taken at the end of the 4-week period. An analysis of epidermal thickness in *in vivo* skin samples by H&E staining showed that retinol and rotational formulations have a significant effect on increasing epidermal thickness when compared to placebo (*p* < 0.05). i: untreated, ii: placebo for retinol, iii: retinol, iv: placebo for PGA, v: PGA, vi: placebo for vii, and vii: rotation of iii and v. Scale bar = 50 μm. *Means *p* < 0.05 or statistically significant result.

**Figure 8:**
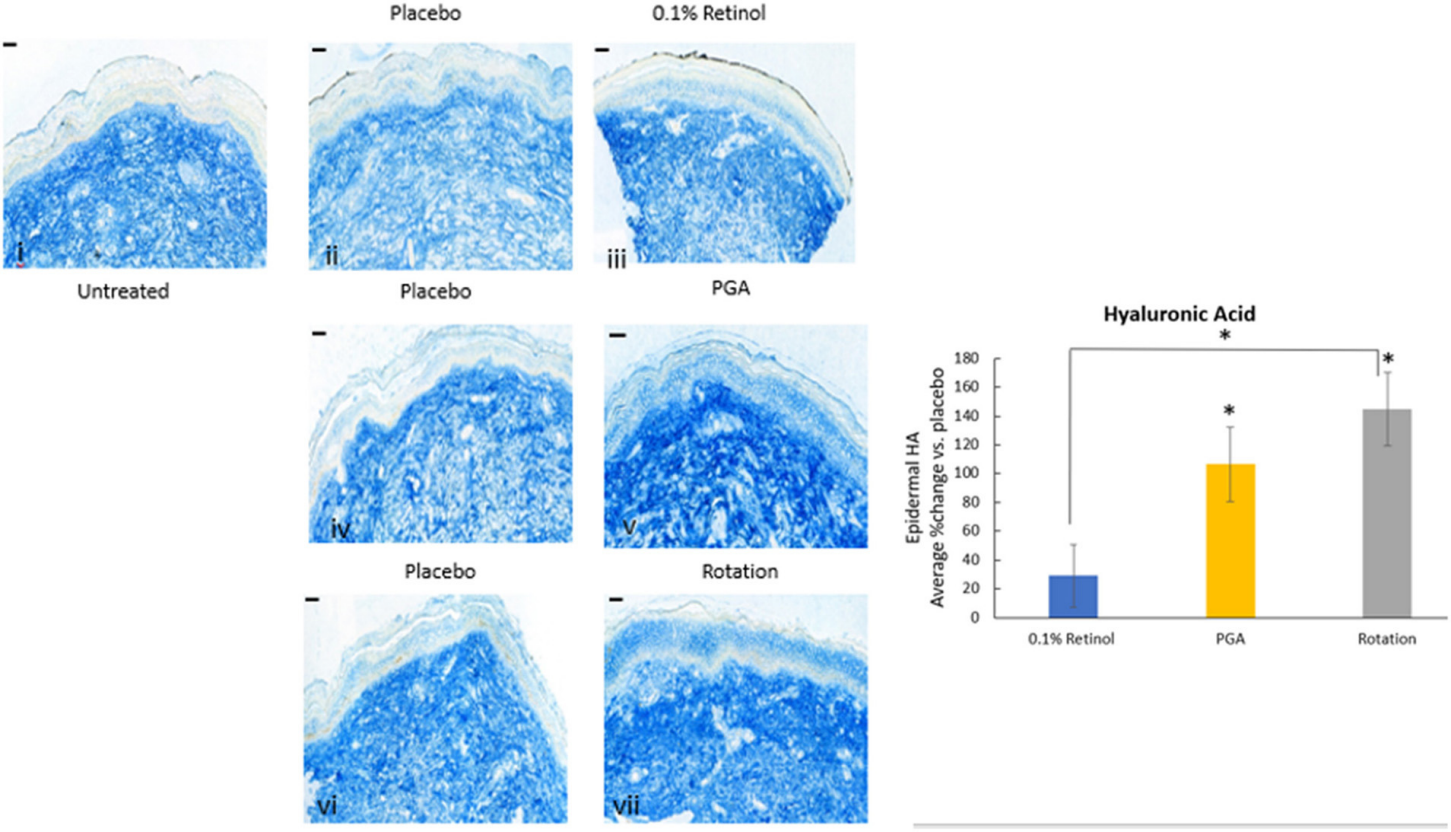
Immunohistology of skin biopsies. Volunteers (*n* = 32) received application of either 0.1% retinol formulation for 4 weeks or weekly rotation of 1% phytol/4% glycolic acid (PGA) formula with 0.1% retinol formula. Biopsy samples were taken at the end of the 4-week period. An analysis of epidermal hyaluronic acid in *in vivo* skin samples *via* HABP staining was performed. Rotational treatment was superior to retinol alone and better than the PGA formula (*p* < 0.05). i: untreated, ii: placebo for retinol, iii: retinol, iv: placebo for PGA, v: PGA, vi: placebo for vii, and vii: rotation of iii and v. Scale bar = 50 μm. *Means *p* < 0.05 or statistically significant result.

**Figure 9:**
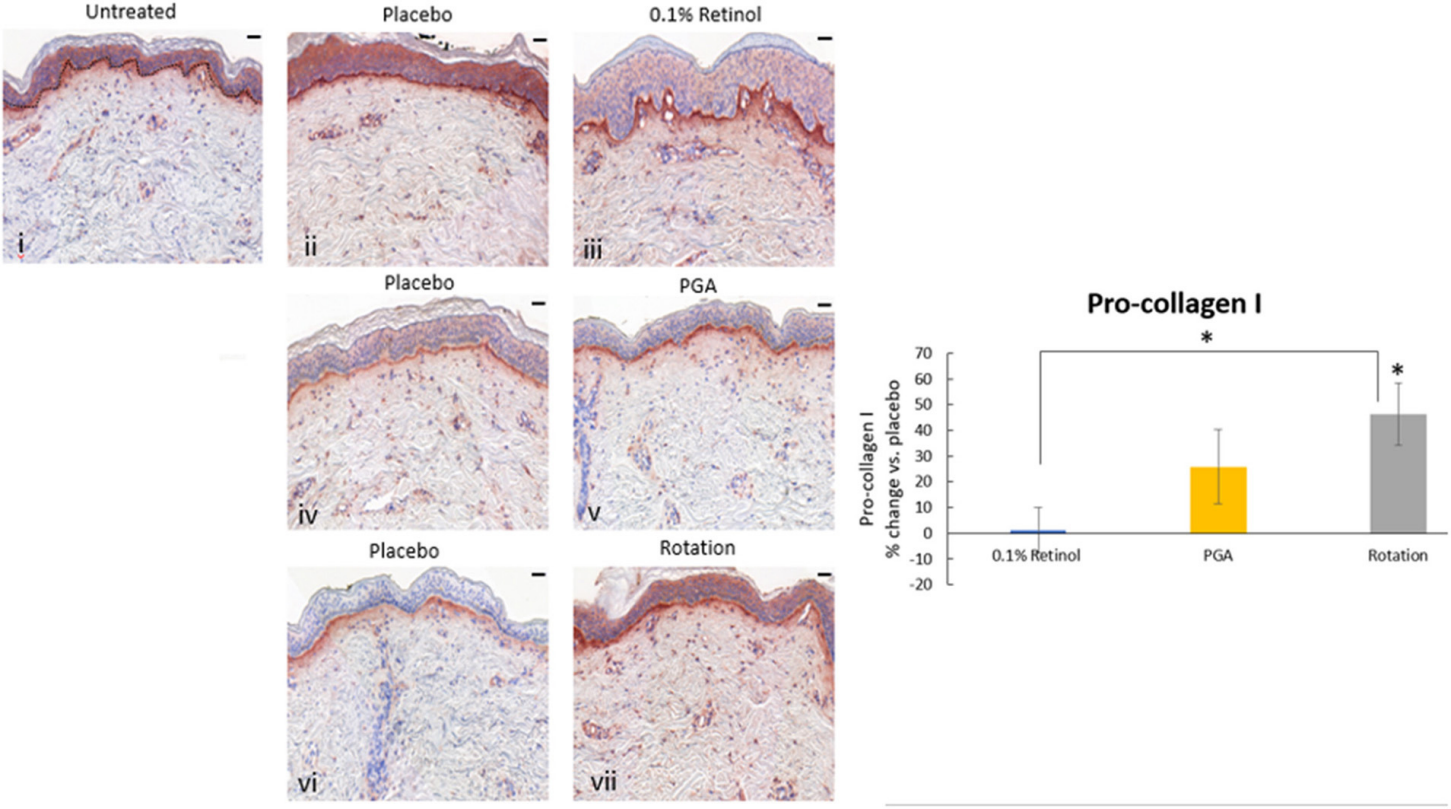
Immunohistology of skin biopsies. Volunteers (*n* = 32) received application of either 0.1% retinol formulation for 4 weeks or weekly rotation of 1% phytol/4% glycolic acid (PGA) formula with 0.1% retinol formula. Biopsy samples were taken at the end of the 4-week period. An analysis of pro-Collagen I in *in vivo* skin samples using pro-collagen I antibody was performed. Pro-collagen I production increased significantly in rotational treatment and was better than retinol alone. i: untreated, ii: placebo for retinol, iii: retinol, iv: placebo for PGA, v: PGA, vi: placebo for vii, and vii: rotation of iii and v. Scale bar = 50 μm. *Means *p* < 0.05 or statistically significant result.

**Figure 10:**
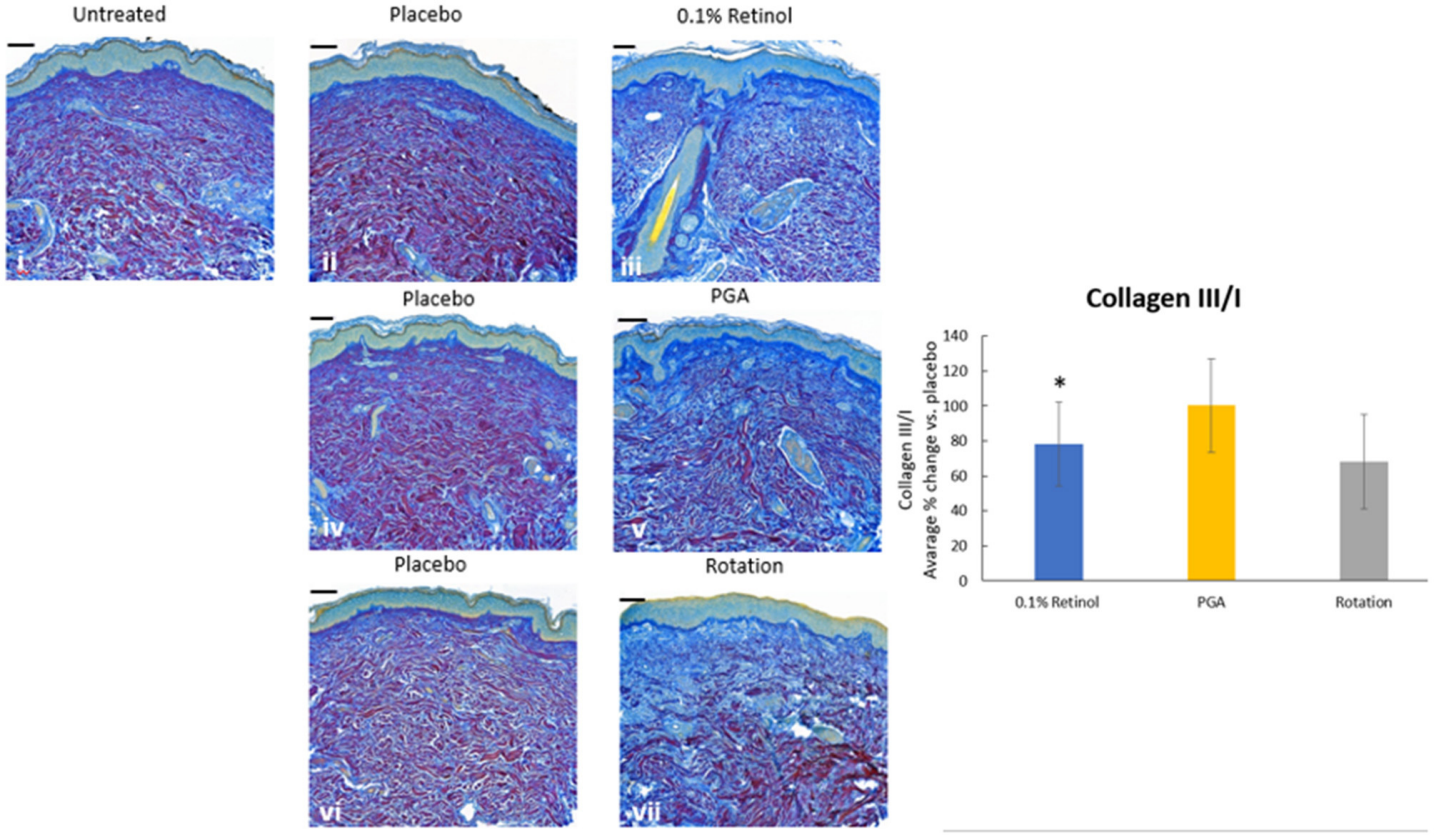
Immunohistology of skin biopsies. Volunteers (*n* = 32) received application of either 0.1% retinol formulation for 4 weeks or weekly rotation of 1% phytol/4% glycolic acid (PGA) formula with 0.1% retinol formula. Biopsy samples were taken at the end of the 4-week period. An analysis of Col III/I ratio in *in vivo* skin samples using Herovici staining was performed to differentiate mature collagen (type I) (pink/red) and immature collagen (type III) (blue). All treatments led to increase in collagen III and improved the ratio of collagen III/I. i: untreated, ii: placebo for retinol, iii: retinol, iv: placebo for PGA, v: PGA, vi: placebo for vii, and vii: rotation of iii and v. *Scale bar = 100 μm.

Formulations were applied to volunteers' (*n* = 32) volar forearm skin under occlusive patch for 4 weeks in a continuous vs. rotational manner. Skin biopsies (2 mm) were analyzed by histology. H&E staining (Figure 7) evaluation showed a significant effect (*p* < 0.01) of retinol and rotational formulations on increasing epidermal thickness when compared to placebo. Of note, rotational treatment was comparable to retinol alone but significantly better (*p* < 0.05) than the PGA formulation. Evaluation of epidermal hyaluronic acid (HA) level using HABP staining (Figure 8) showed an increased expression level of HABP in the epidermal layer after treatment with all formulations, indicative of an increase in HA. Rotational treatment was superior to retinol alone (144.76 vs. 29.07% increase, *p* < 0.05) and better than the PGA formula (106.47% increase, *p* < 0.05). Pro-collagen I was also assessed *in vivo* (Figure 9). Pro-collagen I production increased (*p* < 0.05) in rotational treatment and was significantly better than retinol alone (46.27 vs. 8.92%). Finally, we analyzed the ratio of collagen I and III, in the biopsy samples using Herovici staining (Figure 10). All three treatments increased collagen III (*p* < 0.05) levels significantly, resulting in an increase in collagen III/I ratio.

### Raman Spectroscopy Analysis Shows Active Penetration and Structural Dermal Changes

**Figure 11:**
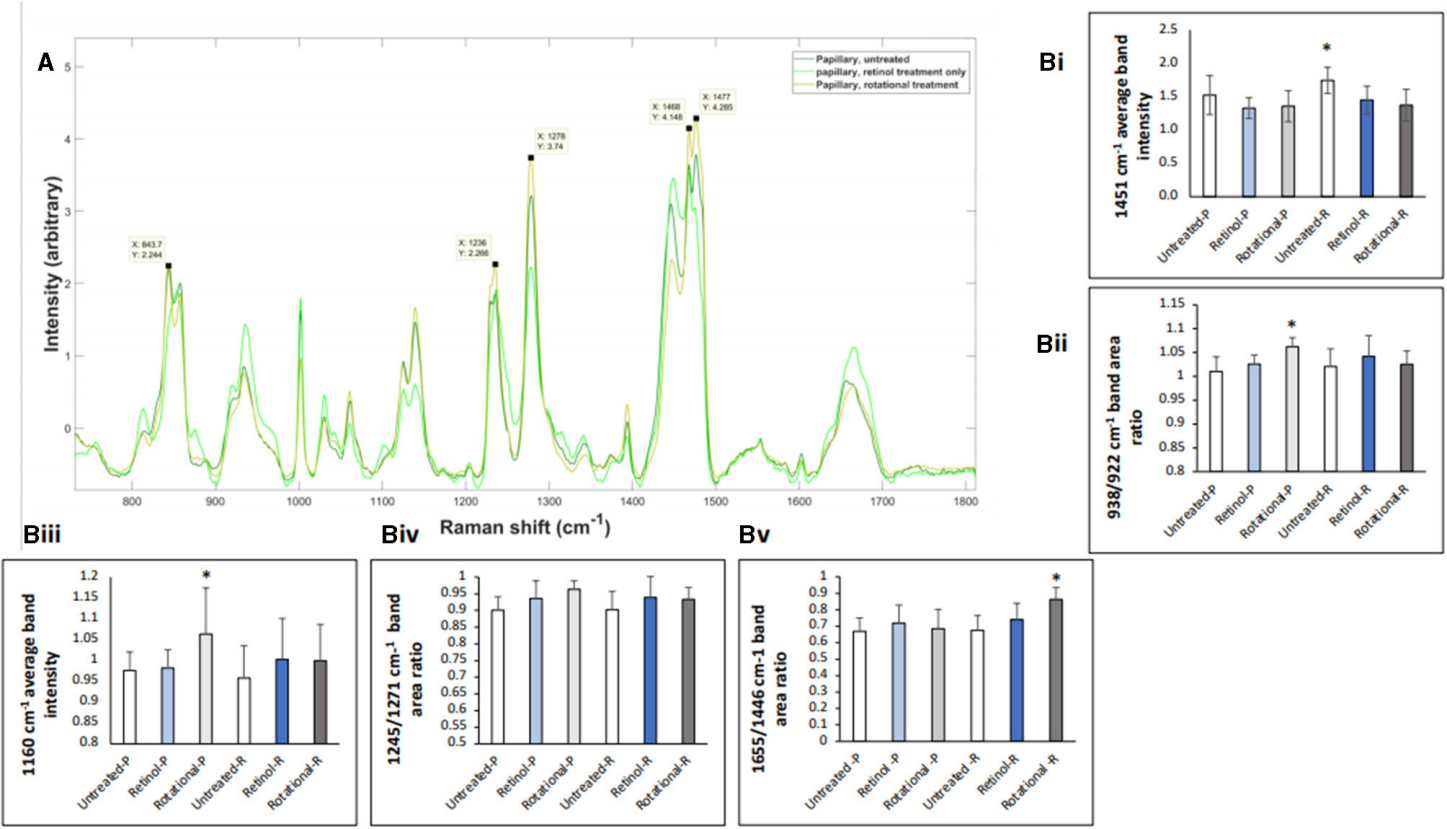
Raman spectroscopy analysis of *in vivo* skin samples. Volunteers (*n* = 32) received application of either 0.1% retinol formulation for 4 weeks or weekly rotation of 1% phytol/4% glycolic acid (PGA) formula with 0.1% retinol formula. Biopsy samples were taken at the end of the 4-week period, and samples were analyzed by confocal Raman. **(A)** Graphs represent the average spectra for all papillary dermis. Key peaks relating to collagen/protein content are labeled as described in the text. **(B)** Bar charts indicating key peaks. (i) The bandwidth 1,451 cm^−1^ represents the CH bending mode of collagen; data collected at this bandwidth was normalized to phenylalanine (Phe) peak at 1,002 cm^−1^. The average band intensity at 1,451 cm^−1^ for untreated, retinol-treated, and rotational-treated samples in the papillary (-P) and reticular (-R) layers is plotted. As the dataset was parametric, a *t*-test was used to demonstrate that there was a significant increase at this bandwidth in untreated samples compared to rotational in the reticular layer. *p < 0.05. (ii) The band area ratio 938/932 cm^−1^ calculated in untreated, retinol-treated, and rotational-treated samples in the papillary (-P) and reticular (-R) layers accounting for proline and hydroxyproline residues of collagen and water, indicating collagen hydration status. All data were standardized to Phe (1,002 cm^−1^). The ratios 938/932 cm^−1^ were tested by one-way ANOVA and then *t*-test was used to evaluate significance (rotational papillary samples significantly greater than untreated). *p < 0.05. (iii) The bandwidth 1,160 cm^−1^ represents glycosaminoglycan (GAG) content; data collected at this bandwidth was normalized to Phe peak at 1,002 cm^−1^. The average band intensity at 1,160 cm^−1^ for untreated, retinol-treated, and rotational-treated samples in the papillary (-P) and reticular (-R) layers is plotted. As the dataset was parametric, a *t*-test was used to demonstrate that there was a significant increase at this bandwidth in rotational papillary samples compared to untreated samples. *p < 0.05. (iv) The band area 1,246/1,271 cm^−1^ ratio calculated in untreated, retinol-treated, and rotational-treated samples in the papillary (-P) and reticular (-R) layers demonstrating the proline poor/proline rich ratio of collagen. The ratios 1,246/1,271 cm^−1^ were tested by one-way ANOVA, and although the trend indicates a higher ratio in retinol and rotational samples in both layers compared to untreated samples, this was not significant, when compared using a *t*-test. Data are standardized to Phe (1,002 cm^−1^). (v) The band area ratio 1,655/1,446 cm^−1^ was calculated in untreated, retinol-treated, and rotational-treated samples in the papillary (-P) and reticular (-R) layers and has also been shown to demonstrate collagen content. The ratios 1,655/1,446 cm^−1^ were tested by one-way ANOVA, and then a *t*-test was used to evaluate significance; rotational samples in the reticular layer had a statistically significant increase in band area ratio compared to untreated samples. Data are standardized to Phe (1,002 cm^−1^). *p < 0.05.

Raman spectroscopy analysis confirmed delivery of treatment active ingredients within the dermis and treatment effects using previously evidenced key peaks in skin biopsies. Figure 11A demonstrates the spectra exhibited through the papillary layer in untreated, retinol-treated, and rotational-treated skin. Some of the bands where the rotational samples have the highest attenuation are highlighted, representing lipids, collagen, and residues of collagen. The band at 1,236 cm^−1^ is well-known to represent amide III, and peaks at 1,271 cm^−1^ are also part of the amide III band but more specifically for proline and hydroxyproline residues of collagen. The peaks at 1,468 and 1,477 cm^−1^ may represent the amide II band. In Figure 11B (i), the band at 1,451 cm^−1^ (the CH bending mode of collagen) was significantly altered throughout untreated samples, compared to rotational treatment (*p* = 0.037). In Figure 11B (ii), the 938:922 cm^−1^ ratio, representing proline and hydroxyproline residues of collagen and water molecules, was significantly increased (*p* = 0.03) in rotational treatment compared to untreated samples in the papillary layer, indicating an increase in collagen hydration status. The glycosaminoglycan (GAG) content assessed at 1,160 cm^−1^ was increased in both treated conditions, with a significantly higher increase (*p* = 0.022) in the rotational samples in the papillary layer (Figure 11B, iii). The proline poor/proline rich ratio 1,246:1,271 cm^−1^ (Figure 11B, iv) demonstrated an increase in retinol and rotational samples compared to untreated samples, although this trend was not significant. The 1,655:1,446 cm^−1^ ratio (Figure 11B, v), representing collagen content, was significantly greater in rotational compared to untreated samples in the reticular dermis (*p* = 0.035).

### Clinical Efficacy Trial of Rotational Treatment Exhibited Incrementally Significant Efficacy Throughout the Year

**Figure 12:**
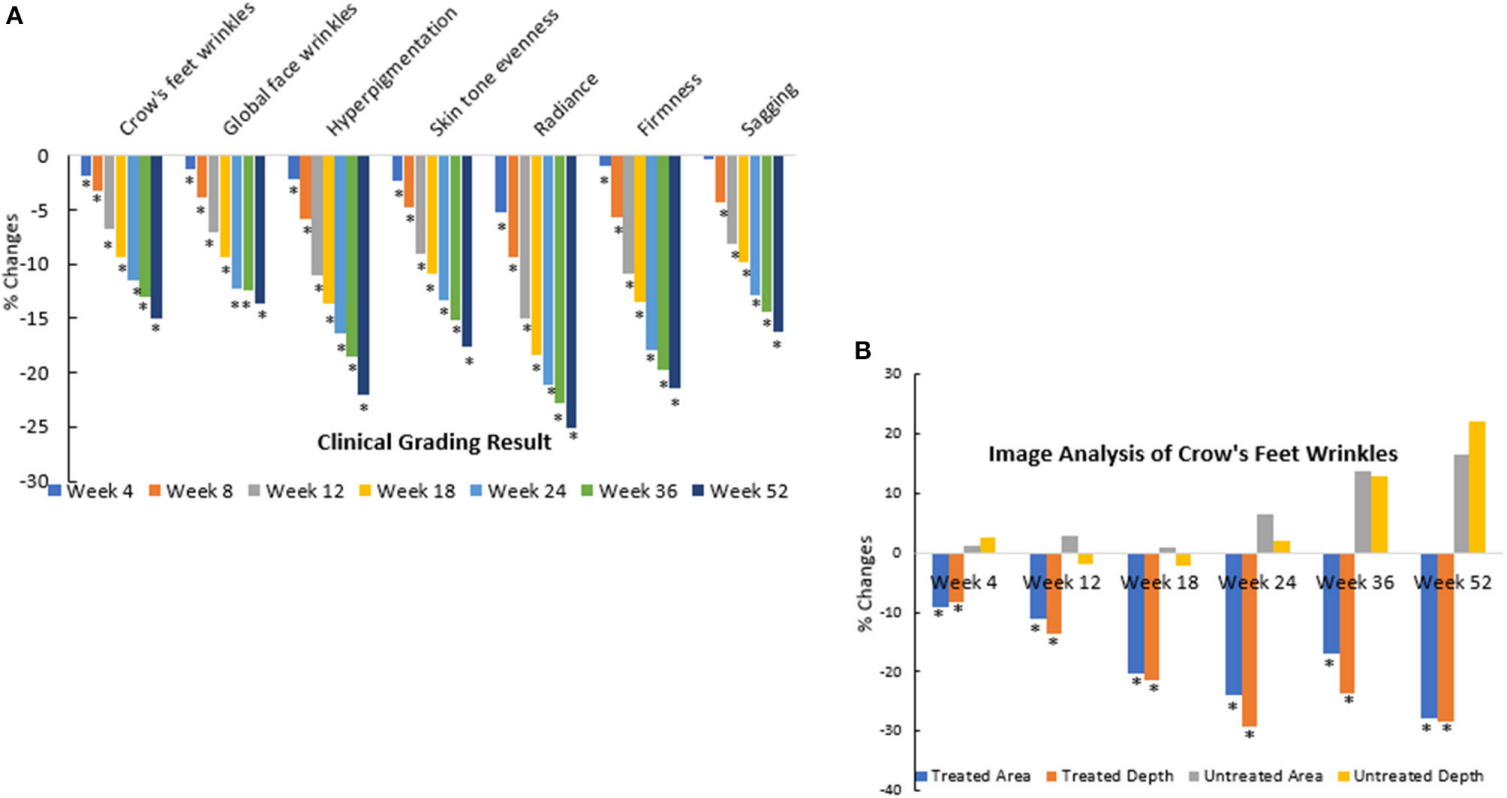
Clinical trial #2 results. **(A)** Dermatological evaluation of 1-year clinical efficacy results. Clinical grading results show reduction (improvement) in photoaging parameters at different timepoints over a 1-year period when compared to baseline scores (*p < 0.05). Statistical significance was achieved for most parameters at week 4 and for all parameters starting from week 8. Note that the improvement was gradual and continuous over the course of the year. **(B)** Image analysis of 1-year clinical efficacy results on crow's feet wrinkles. Image analysis shows reduction (improvement) in wrinkle parameters at the crow's feet area at different timepoints over a 1-year period when compared to baseline values (*p < 0.05). Note that the untreated side showed worsening over time.

**Figure 13:**
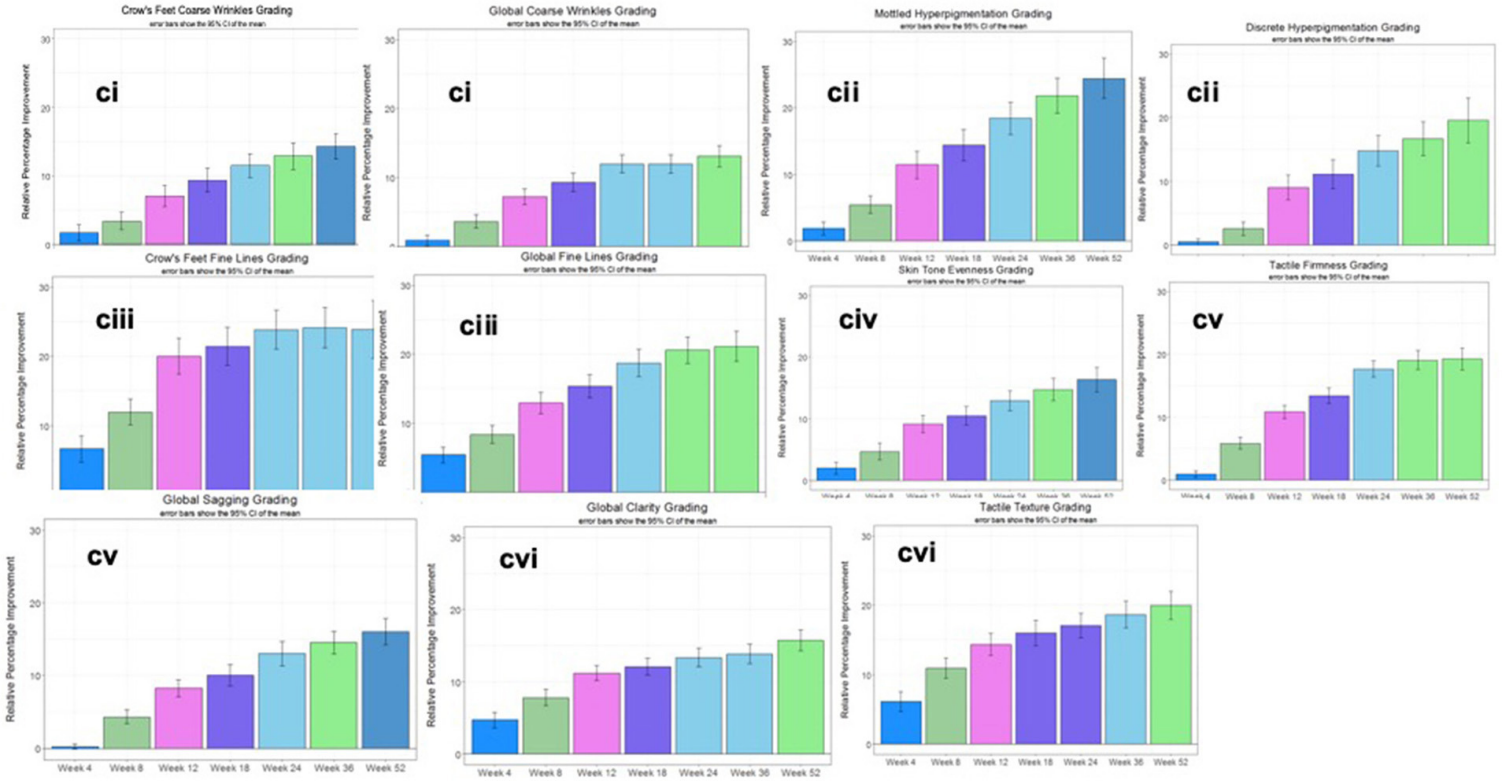
Clinical trial #2 results. (Ci) Month-to-month significant improvement in dermatological grading up to 1 year. Dermatological grading shows statistically significant improvement (*p* < 0.05) in global crow's feet coarse wrinkle parameters. Bar color changes month-to-month indicates statistical improvement from previous timepoint. This pattern of improvements noted throughout the year are an indication the plateau effect can be overcome with this specific rotational formulation. (Cii) Month-to-month significant improvement in dermatological grading up to 1 year. Dermatological grading shows statistically significant improvement (*p* < 0.05) in mottled and discrete hyperpigmentation parameters. Bar color changes month to month indicates statistical improvement from previous timepoint. This pattern of improvements noted throughout the year are an indication the plateau effect can be overcome with this specific rotational formulation. (Ciii) Month-to-month significant improvement in dermatological grading up to 1 year. Dermatological grading shows statistically significant improvement (*p* < 0.05) in global fine and crow's feet wrinkle parameters. Bar color changes month to month indicates statistical improvement from previous timepoint. This pattern of improvements noted throughout the year are an indication the plateau effect can be overcome with this specific rotational formulation. (Civ) Month-to-month significant improvement in dermatological grading up to 1 year. Dermatological grading shows statistically significant improvement (*p* < 0.05) in skin tone evenness parameter. Bar color changes month to month indicates statistical improvement from previous timepoint. This pattern of improvements noted throughout the year are an indication the plateau effect can be overcome with this specific rotational formulation. (Cv) Month-to-month significant improvement in dermatological grading up to 1 year. Dermatological grading shows statistically significant improvement (*p* < 0.05) in tactile firmness and global sagging parameters. Bar color changes month to month indicates statistical improvement from previous timepoint. This pattern of improvements noted throughout the year are an indication the plateau effect can be overcome with this specific rotational formulation. (Cvi) Month-to-month significant improvement in dermatological grading up to 1 year. Dermatological grading shows statistically significant improvement (*p* < 0.05) in global clarity and tactical texture parameters. Bar color changes month to month indicates statistical improvement from previous timepoint. This pattern of improvements noted throughout the year are an indication the plateau effect can be overcome with this specific rotational formulation.

The second clinical trial was designed to evaluate the efficacy of the rotational skin care regimen for 1 year to determine if this regimen would deliver continued improvement over the course of the year. A total of 116 female subjects completed the trial (Table 3). Clinical evaluation by expert graders showed that treatment with the rotational regimen resulted in statistically significant improvement in all photoaging parameters after as few as 4 weeks of product usage (Figure 12A). The improvement was continuous throughout the course of the year, with 10–40% subjects showing improvement of various parameters at week 4 and up to 90–100% subjects showing improvement in all parameters at year end. The mean percentage change of the whole population also increased from 1–5% at week 4 to 15–25% at year end. The untreated side of the face showed minimal improvement, with hyperpigmentation getting worse (with a mean change of 1–2% on key photoaging parameters: coarse wrinkles and hyperpigmentation). Image analysis of crow's feet wrinkles/lines provided objective and quantitative assessment to support the clinical findings (17). Wrinkle condition in the crow's feet area was quantified for wrinkle count, length, width, perceived depth, and area (Figure 12B). All parameters showed statistically significant improvement (*p* < 0.05) on treated side with rotational regimen and no improvement or worsening on untreated side. In addition, most parameters exhibited statistically significant improvement between each consecutive timepoint evaluated (Figure 13, ci–vi).

**Table 3:** Demographic information on clinical trial #2.

	**All subjects**	
***N***	130	
**Age (years)**		
Mean	58.7 ± 8.0	
**Ethnicity**				
Hispanic or Latino	17	(13.1)
Not Hispanic or Latino	113	(86.9)
**Race**		
American Indian or Alaska Native	1	(0.8)
Asian	16	(12.2)
Black or African American	0	(0.0)
White	112	(86.2)
Multiracial	1	(0.8)
**ITT population**	130	
**Completed subjects (PP population)**	116	
**Discontinued subjects**	14	
**Reason for discontinuation**		
Adverse event	9	
Non-compliance	2	
Investigator decision	1	
Lost to follow-up	2	

*ITT, Intend-to-Treat; PP, Per Protocol*.

### Clinical Efficacy Trial of Rotational Treatment Was Significantly Better Than Similar Antiaging Products

The third trial was designed to compare rotational combination regimen against competitive products. Two products were chosen: one with retinol alone (product C) and one with antioxidant and peptides purported to boost collagen production (product D). A total of 144 subjects completed the third clinical trial. Subjects were randomized into two groups, 1 and 2. Both groups used the rotational regimen alongside either of the marketed products, C (those in group 1) or D (group 2). Demographic information is listed in Table 4.

**Table 4:** Demographic information on clinical trial #3.

	**Group 1: rotational product vs. product C**		**Group 2: rotational product vs. product D**	
***N***	83		81	
**Age (years)**				
Mean	58.9 ± 8.2		59.6 ± 7.1	
**Ethnicity**					
Hispanic or Latino	4	(4.8)	6	(7.4)
Not Hispanic or Latino	79	(95.2)	75	(92.6)
**Race**					
American Indian or Alaska Native	0	(0.0)	1	(1.2)
Asian	11	(13.3)	4	(4.9)
Black or African American	7	(8.4)	12	(14.9)
White	64	(77.1)	63	(77.8)
Multiracial	1	(1.2)	1	(1.2)
**ITT population**	83		81	
**Completed subjects (PP population)**	74		70	
**Discontinued subjects**	9		11	
**Reason for discontinuation**					
Adverse event	1		5	
Subject requested withdrawal	4		2	
Non-compliance	0		2	
Investigator decision	1		1	
Lost to follow-up	3		1	

*ITT, Intend-to-Treat; PP, Per Protocol*.

**Figure 14:**
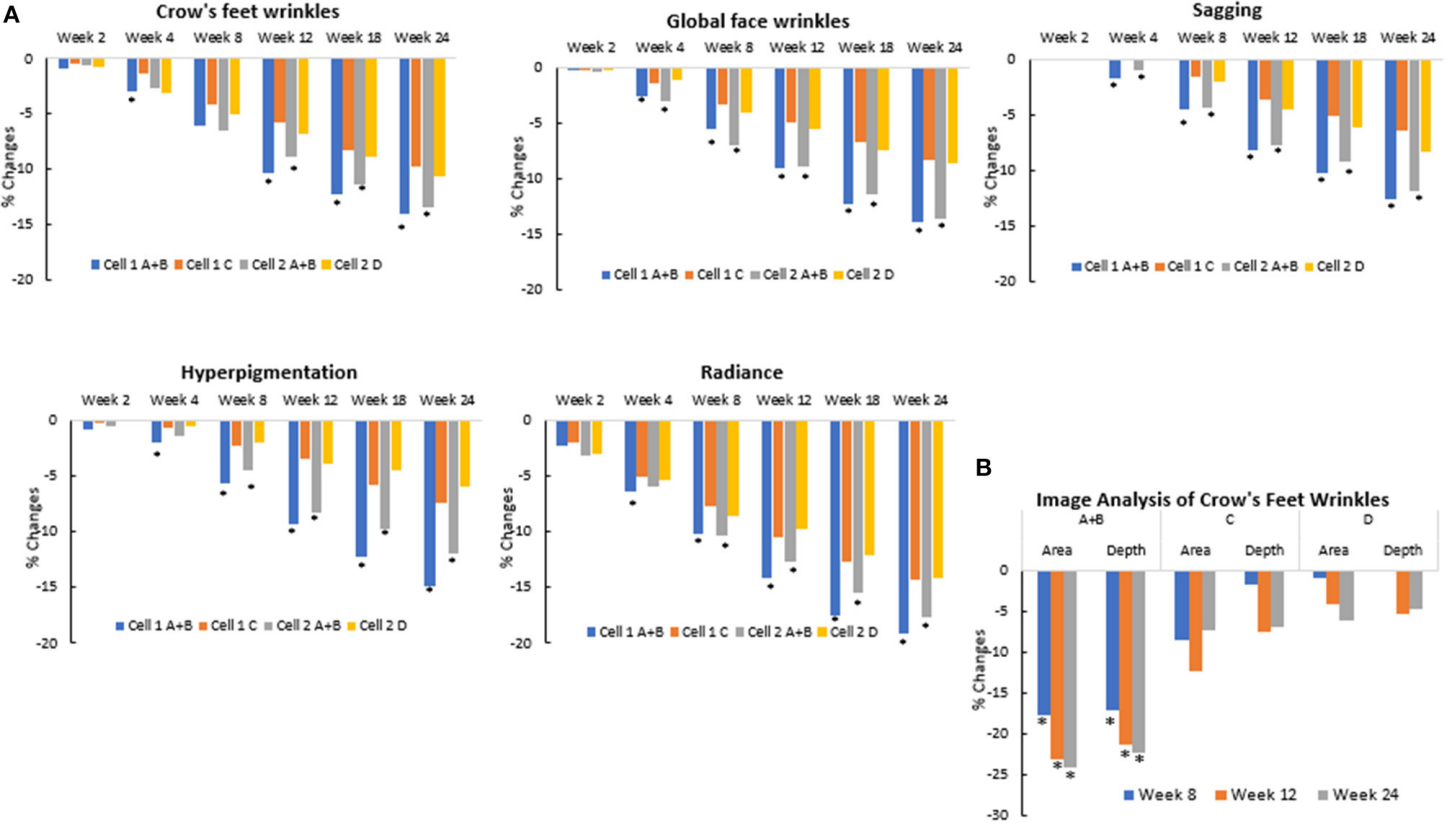
Clinical trial #3 results. **(A)** Clinical efficacy study results of rotational treatment vs. two commercial products. Clinical grading results show reduction (improvement) in photoaging parameters at different timepoints when compared to baseline scores and when the rotational regimen (A + B) is compared to marketed products (C or D). An asterisk indicates a statistically significant difference between the rotational regimen and product C or D (*p* < 0.05). **(B)** Crow's feet wrinkle image analysis of clinical efficacy study results of rotational treatment vs. two commercial products. Image analysis result shows reduction (improvement) in wrinkle parameters at the crow's feet area at different timepoints when compared to baseline values. Differences between the rotational regimen (A + B) vs. marketed products (C or D) were statistically significant at all three time points shown here (*p < 0.05).

All products produced significant improvement on various facial photoaging signs over the course of the study (*p* ≤ 0.05) vs. marketed products and baseline. However, in both groups, the rotational products outperformed the marketed products C and D (Figure 14A). For some parameters, the difference was seen as early as week 4; by week 12, all parameters showed greater improvement on the side treated with the rotational products than the side with the marketed products. These findings were corroborated by image analysis. The side treated with the rotational products displayed a significant (*p* ≤ 0.05) reduction in crow's feet wrinkles/lines than the side treated with marketed products C or D (Figure 14B).

## Discussion

The hypothesis that applying multiple antiaging ingredients in a rotational or alternating regimen to address facial photodamage signs originates from our confirmed theory that the effect on skin of a single active ingredient, such as retinol, may plateau over time. Therefore, this hypothesis was systematically tested in a multi-tiered approach using *in vitro, ex vivo*, and *in vivo* studies. Results presented herein show that a decreasing rate of improvement, or the plateau effect, can indeed be reduced with a novel “rotational” regimen of active ingredients.

Retinol is a well-accepted, efficacious ingredient (23–25) shown to have long-term efficacy (26); however, sustaining the rate of improvement has not been demonstrated. In order to identify a more effective treatment approach, active ingredients were tested in different application regimens *in vitro*. We found that a sequential application of phytol and retinol stimulated a higher-level production of collagen from human dermal fibroblasts. PGA and retinol pair was further confirmed *ex vivo*. One reason for this enhancement in efficacy may be that retinol acts in skin after conversion to retinoic acid, which in turn activates retinoid X receptor (RXR) and retinoic acid receptor (RAR) (27), while phytol is converted to phytanic acid, which has been shown to be a ligand of not only RXR but also peroxisome proliferator-activated receptors (PPARs) (28). Due to their receptor binding specificities, retinol and phytol can be viewed as not only competing but also complementary ingredients. Although glycolic acid can provide an additional skin barrier and renewal benefit (29), it is difficult to formulate together with retinol due to specific formula pH restrictions (unpublished own data). Therefore, glycolic acid was added to the phytol formula in order to obtain the maximum possible efficacy and benefit from all three ingredients.

When the effect of those formulations on human skin were assessed *in vivo* through patch application followed by skin biopsy, rotational application of a PGA-based formula and a retinol formula showed enhanced or at least similar results compared to continuous single treatment as measured by multiple skin markers. The biopsies used for RS analysis showed the effect of rotational actives on dermal structure post-application. Indeed, subtle changes were recognizable within each layer of the treated skin samples, as demonstrated by alterations in relevant peak formation and attenuation. Band assignments to these changes and ratios allowed for correlation with clinical and histological findings (30–32), demonstrating significant increases in residues of collagen and collagen content for the rotational treatment compared to untreated samples. Interestingly, the band at 1,160 cm^−1^ representing GAG content was also significantly increased in the rotational samples compared to the untreated group. These findings further corroborate histological increases in pro-collagen I and HABP.

Clinical testing of the aforementioned regimen *in vivo* used a weekly rotational application of a PGA-based formula and a retinol-based formula. Two randomized, double-blind, IRB-approved, controlled clinical trials were conducted to test the efficacy of this regimen. The application of the rotational cosmetic regimen over the course of 1 year resulted in continued improvement in facial photoaging signs without a plateau effect. Comparing the rotational regimen to marketed products, the rotational regimen sustained greater improvement over the course of 6 months and showed long-lasting benefits during the 2-month regression period.

Our findings here, for the first time, identify a novel approach for a skin regimen that can produce long-lasting antiaging effects. Our rotational application strategy has some advantages over combination treatments alone including continued enhanced efficacy throughout the 12-month study duration. It avoids the need to make a single product containing all active ingredients, as some may not be compatible for formulation or lead to undesirable side effects. It also simplifies product usage each day to one product, although keeping track of product rotation may require help from additional application, packaging, or delivery apparatus. Overall, this strategy may provide a faster, more effective venue for new developments in cosmetic antiaging products.

## Data Availability Statement

The raw data supporting the conclusions of this article will be made available by the authors, without undue reservation.

## Ethics Statement

The studies involving human participants were reviewed and approved by IRB-approved (IntegReview, Austin, TX). The patients/participants provided their written informed consent to participate in this study.

## Author Contributions

All authors listed have made a substantial, direct and intellectual contribution to the work, and approved it for publication.

## Funding

This study was funded by Avon Products, Inc. Employees of the sponsor were involved in study design. AB acknowledges support from the NIHR Manchester Biomedical Research Center based at the University of Manchester, Manchester, England, UK as well as the MRC (SA) for funding Hair and Skin Research Laboratory, Division of Dermatology, Groote Schuur Hospital, the University of Cape Town, South Africa.

## Conflict of Interest

LD, JI-B, YZ, and AG are all employees of Avon Skin Care Institute, Global Innovation Center, Avon Products Inc., Suffern, NY, USA. TS and LJ are employees of Thomas J. Stephens & Associates and received study funding from Avon, USA. RB is a postgraduate student at the University of Manchester. WL and AB are employees of the University of Manchester, Manchester, England. University of Manchester, which received funding from Avon for conducting this study, approved study.

## Publisher's Note

All claims expressed in this article are solely those of the authors and do not necessarily represent those of their affiliated organizations, or those of the publisher, the editors and the reviewers. Any product that may be evaluated in this article, or claim that may be made by its manufacturer, is not guaranteed or endorsed by the publisher.
